# HIV Viremia Is Associated With *APOL1* Variants and Reduced JC-Viruria

**DOI:** 10.3389/fmed.2021.718300

**Published:** 2021-08-27

**Authors:** Etty Kruzel-Davila, Barbara Mensah Sankofi, Ernestine Kubi Amos-Abanyie, Anita Ghansah, Alexander Nyarko, Seth Agyemang, Gordon A. Awandare, Moran Szwarcwort-Cohen, Anat Reiner-Benaim, Basem Hijazi, Ifeoma Ulasi, Yemi Raheem Raji, Vincent Boima, Charlotte Osafo, Victoria May Adabayeri, Michael Matekole, Timothy O. Olanrewaju, Samuel Ajayi, Manmak Mamven, Sampson Antwi, Adebowale D. Ademola, Jacob Plange-Rhule, Fatiu Arogundade, Priscilla Abena Akyaw, Cheryl A. Winkler, Babatunde L. Salako, Akinlolu Ojo, Karl Skorecki, Dwomoa Adu

**Affiliations:** ^1^Rappaport Faculty of Medicine, Technion Israel Institute of Technology, Haifa, Israel; ^2^Nephrology Department, Rambam Health Care Campus, Haifa, Israel; ^3^College of Health Sciences, Noguchi Memorial Institute for Medical Research, University of Ghana, Accra, Ghana; ^4^West African Centre for Cell Biology of Infectious Pathogens, College of Basic and Applied Sciences, University of Ghana, Accra, Ghana; ^5^University of Ghana Medical School, College of Health Sciences, University of Ghana, Accra, Ghana; ^6^Rambam Health Care Campus, Haifa, Israel; ^7^Azrieli Faculty of Medicine, Bar-Ilan University, Safed, Israel; ^8^Department of Medicine, College of Health Sciences University of Nigeria, Enugu, Nigeria; ^9^Department of Medicine, University of Ibadan, Ibadan, Nigeria; ^10^Department of Medicine, Kwame Nkrumah University of Science and Technology, Kumasi, Ghana; ^11^Department of Medicine, University of Abuja, Abuja, Nigeria; ^12^Obafemi Awolowo University, Ile-Ife, Nigeria; ^13^Department of Medicine, Frederick National Laboratory for Cancer Research (NIH), Frederick, MD, United States; ^14^School of Medicine, University of Kansas Medical Center, Kansas City, KS, United States

**Keywords:** *APOL1*, HIV viremia, JC viruria, BK viruria, innate immune, kidney disease

## Abstract

Variants in the *Apolipoprotein L1* (*APOL1*) gene (G1-rs60910145, rs73885319, G2-rs71785313) are common in Africans and in individuals of recent African ancestry and are associated with an increased risk of non-diabetic chronic kidney disease (CKD) and in particular of HIV associated nephropathy (HIVAN). In light of the significantly increased risk of HIVAN in carriers of two *APOL1* risk alleles, a role in HIV infectivity has been postulated in the mechanism of *APOL1* associated kidney disease. Herein, we aim to explore the association between HIV viremia and *APOL1* genotype. In addition, we investigated interaction between BK and JC viruria, CKD and HIV viremia. A total of 199 persons living with HIV/AIDS (comprising 82 CKD cases and 117 controls) from among the participants in the ongoing Human Heredity and Health in Africa (H3Africa) Kidney Disease Research Network case control study have been recruited. The two *APOL1* renal risk alleles (RRA) genotypes were associated with a higher risk of CKD (OR 12.6, 95% CI 3.89–40.8, *p* < 0.0001). Even a single APOL1 RRA was associated with CKD risk (OR 4.42, 95% CI 1.49–13.15, *p* = 0.007). The 2 APOL1 RRA genotypes were associated with an increased probability of having HIV viremia (OR 2.37 95% CI 1.0–5.63, *p* = 0.05). HIV viremia was associated with increased CKD risk (OR 7.45, 95% CI 1.66–33.35, *P* = 0.009) and with a significant reduction of JC virus urine shedding (OR 0.35, 95% CI 0.12–0.98, *p* = 0.046). In contrast to prior studies, JC viruria was not associated with CKD but was restricted in patients with HIV viremia, regardless of CKD status. These findings suggest a role of *APOL1* variants in HIV infectivity and emphasize that JC viruria can serve as biomarker for innate immune system activation.

## Introduction

Two variants at the *APOL1* gene, encoding Apolipoprotein L1, account for more than 70% of the increased risk for non-diabetic chronic kidney disease (CKD) in individuals of African ancestry (1–3). The ancestral non-renal risk allele has been designated G0 and the two risk alleles (renal risk alleles, RRA) have been designated as G1 (encoding S342G and I384M substitutions) and G2 (encoding the combined N388 and Y389 deletions), respectively (1, 3). These variants have risen to high allele frequency due to a dominant selective advantage that restores trypanolytic activity against *T. b. rhodesiense* (G2) and confers protection from active illness caused by *T. b. gambiense* (G1) (1, 4–6). Unexpectedly, the G2 genotype is associated with active *T. b. gambiense* infection (5). It is an enigma why some of the highest frequencies of G2 are also found in regions of West Africa where *T. b. gambiense* is endemic, despite the observed G2 association with symptomatic chronic sleeping sickness. This raises the likelihood of a broader role in pathogen resistance by the RRA, extending beyond trypanosomes (4–6). Therefore, the protective role of *APOL1* variants in various infectious diseases is under continued extensive investigation. HIV infection deserves special consideration in regard to modulation of HIV infectivity by *APOL1* variants. In light of the significantly increased risk of HIV associated nephropathy (HIVAN) in carriers of two *APOL1* RRA (2, 7), a role in HIV infectivity has been postulated. *In vitro* studies have demonstrated that G0 APOL1 restricts HIV replication in macrophages and differentiated monocytes (8). APOL1 was also identified as one of the HIV restriction proteins, by using genome-wide scans for human genes sharing molecular and evolutionary signatures of known restriction factors. These findings were also validated using *in vitro* studies demonstrating anti-HIV- activity (9). In addition, the presence of RRA but not G0 *APOL1* led to persistence of HIV infection in human podocytes in synergy with IL-1beta (10). Recently, a genetic association study (ALIVE - AIDS Link to the Intravenous Experience cohort) revealed no evident association of *APOL1* RRA with HIV infection acquisition, viral load or disease progression, in antiretroviral therapy (ART) naïve patients (11). That study examined HIV natural history cohorts enrolling African Americans prior to the ART era. The large number of treatment-naïve seroconverters made it a choice cohort for unbiased exploration of HIV-related outcomes. Herein, we explored the association of HIV viremia and CKD with *APOL1* variants in participants recruited from HIV clinics in Africa. All the participants were attending HIV clinics were likely to have received ART, but we cannot be certain of adherence. We also examined the association between JC and BK viruria in all participants, in light of previous findings demonstrating reduced JC viruria in CKD patients and the expected increased BK urine shedding in immunocompromised HIV infected patients (12–17).

## Materials and Methods

### Ethics Statement

Ethical approval for the study was obtained from all the Institutional Review Boards of each participating institution as well as the lead clinical center at the University of Ghana (Ghana Health Service Ethics Review Committee) who approved the study protocols. Written informed consent was obtained from all study participants.

### Study Participants

Age and sex matched Africans from Ghana and Nigeria with HIV infection were recruited as part of the H3Africa Kidney Disease Research Network, from 2013 to 2018.

The study group comprises 82 individuals with CKD and 117 individuals with normal kidney function. The estimated glomerular filtration rate (eGFR) was computed using the creatinine-based Chronic Kidney Disease Epidemiology Collaboration equation without race adjustment (CKD-EPI) (18). CKD was defined as eGFR <60 mL/min/1.73m^2^, or eGFR more than 60 ml/min/1.73m^2^ with albumin to creatinine ratio (ACR) > 2.5 mg/mmol (male) > 3.5 mg/mmol (female). Control participants enrolled had eGFR > 90 ml/min and ACR <2.5 mg/mmol (male), <3.5 mg/mmol (female). HIV viral load was examined at enrollment.

### HIV Viral Load

Plasma HIVRNA quantitative analysis was assayed as previously reported by Tung et al. using the Roche Cobas Ampliprep/Taqman qRT-PCR assay, v. 3.1.2 Roche Diagnostics, Indianapolis, IN, USA (19). The test quantitates HIV RNA over the range of 20–10,000,000 copies/mL.

### Genotyping of *APOL1* G1-G2 Risk Variants

*APOL1* renal risk variants G1 (rs73885319, p.S342G and rs60910145, p.I384M) and G2 (rs71785313, p.N388_Y389del) were genotyped by custom TaqMan genotyping assays (Applied Biosystems, Foster City, CA) (20).

### Nucleic Acid Extraction and Quantitative PCR for JCV and BKV

Frozen urine (200 mL) samples from the participants were thawed, and total nucleic acid was extracted. The extraction procedure used the Magna Pure LC, version 2.0, apparatus and Magna Pure LC total Nucleic Acid Isolation Kit Reagents (Roche Diagnostics, Germany) according to the manufacturer's instructions (elution volume, 100 mL).

### TaqMan Real Time PCR

The primers and probes were derived from nucleotide sequences of the N terminus of the viral T antigen of JCV and BKV: JCV, 5′-GAGTGTTGGGATCCTGTGTTTT-3′ (forward); 5′ -AGAAGTGGGATGAAGACCTGTTT-3′ (reverse); and Probe 5′FAM-TCATCACTGGCAAACATTTCTTCATGGC- BHQ-1-3′; BKV: 5′-TTGCTTCTTCATCACTGGCAA-3′ (forward); 5′-AGTCCTGGTGGAGTTCCTTTAATG-3′ (reverse); and probe 5′FAM-CATATCTTCATGGCAAAATAAATCTTCATCTCATCCCATTT-BHQ-1-3′.

The PCR reaction was performed in a total volume of 25 ul containing Absolute Blue quantitative PCR mix (Thermo Scientific, UK) in the presence of 10 mL target DNA, 300 nM of each primer and 200 nM of the probe. PCR was performed using the rotor gene 6000/Q instrument (Corbett Research/Qiagen, Hilden, Germany) under the following conditions: 15 min at 95°C and 45 cycles of 15 s at 95°C and 60 s at 60°C. For quantitative results analysis, an average standard curve was constructed using quantified JCV and BKV DNA (Advanced Biotechnology Industry, MD). The results are reported as the number of JCV/BKV genome copies per 1 mL of urine. The lowest detection level was 100 genomic copies per 1 mL.

### Statistical Analysis

Univariate analysis (Chi-squared test, *t*-test) was used to compare case and control groups. Multivariate logistic regression with stepwise, forward and backward model selection was used to predict the risks for HIV viral load, JC and BK viruria and CKD. Each covariate was corrected for all other variables. The covariates that were included for CKD were: age, gender, APOL1 RRA, BK viruria, JC viruria and HIV viremia. The interaction between APOL1 RRA and HIV viremia was added to the CKD risk association model. For CKD risk, we adjusted sequentially to four models: model 1: for *APOL1* variants, age and sex, model 2: further adjusted for BK and JC viruria, model 3: as for model 2 plus HIV viremia and model 4: as for model 3 plus the interaction of APOL1 RRA and HIV viremia. For models predicting HIV viremia, JC and BK viruria, the following covariates were included in the logistic regression: age, gender, CKD, urine albumin/creatinine, APOL1 RRA and the other two viruses, respectively.

## Results

### Association With CKD

The demographic and clinical characteristics for participants in this cohort, stratified by case-control status (*n* = 82, *n* = 117, respectively) are presented in Tables 1, 2. The CKD patient group and the control group did not differ significantly by age (*p*-value = 0.06) or sex (*p*-value = 0.154) (Table 1). We compared the distribution of G1 and G2 RRAs in HIV seroprevalent subjects with and without CKD. The following covariates were included: age, gender, APOL1 RRA, HIV viremia, BK viruria, JC viruria and the interaction 0f HIV^*^ APOL1 RRA. Multivariate logistic regression with a stepwise, forward and backward model selection showed that even a single APOL1 RRA was associated with CKD, OR 4.429 (95% CI 1.49–13.15, *p* = 0.007). As expected, 2 APOL1 RRA were significantly associated with CKD, OR 12.6 (95% CI 3.89–40.80, *p* < 0.0001). HIV viremia was also associated with CKD, OR 7.45 (95% CI 1.66–33.35, *p* = 0.009). The interaction between APOL1 RRA and HIV was significant (*p* = 0.011), with OR 0.081 and 0.073 with 1 and 2 APOL1 RRA, respectively. This reverse association is perplexing and might be explained by Simpson's paradox, and does not seem to confer biological significance (21–23) (Table 3). Interestingly, adjusted models for age, sex, BK, JC and HIV viremia (models 2 and 3, respectively) did not change the association of 2 APOL RRA with CKD while adjustment for APOL1 RRA^*^HIV interaction (model 4) increased the OR for 1 and 2 APOL1 RRA significantly (Table 4).

**Table 1:** Comparison of CKD patients and control.

		**Controls (** ***n*** **=** **117)**		**CKD (** ***n*** **=** **82)**		
		**Mean**	**SD**	**Mean**	**SD**	***P*-value**
Age		41.89	9.58	44.38	9.57	0.060
Log_HIV load		2.79	1.26	2.98	1.36	0.698
Log BK viruria		2.46	1.55	2.92	1.55	0.200
Log_JC viruria		4.46	1.57	4.35	1.80	0.610
		***n***	**%**	***n***	**%**	
Gender	Male	33	28.2%	31	37.8%	0.154
APOL1 risk alleles Copies	0	39	33.3%	11	13.4%	<0.001
	1	51	43.6%	31	37.8%	
	2	27	23.1%	40	48.8%	
HIV	pos	34	29.1%	28	34.1%	0.446
BK	pos	26	22.2%	20	24.4%	0.721
JC	pos	22	18.8%	13	15.9%	0.591

**Table 2:** Demographic and clinical characteristics for individuals participating in this cohort, stratified by case-control status.

**Baseline demographic characteristics and clinical parameters**				
	**HIV** **+Cases with CKD**		
	**APOL1 -**	**APOL1 -**	**APOL1 -**	**APOL1 -**
	**0 risk alleles (11)**	**1 risk alleles (31)**	**0** **+** **1 risk alleles (42)**	**2 risk alleles (40)**
Age at enrollment, y	39.8 (35.8–44.8)	46.2 (40.9– 51.1)	44.8 (38.9– 50.8)	44.8 (36.5–51.5)
Female sex, *n* (%)	63.6	67.7	66.6	62.9
CKD-EPI eGFR, mL/min/1.73 m^2^	42.7 (20.1–51.2)	36.8 (22.3–56.2)	39.3 (20.6–54.9)	18.6 (11.6–42.3)
UACR, mg/mmol	1.5 (0.5–2.4)	2.79 (0.99–10.7)	2.26 (0.94–6.25)	3.2 (0.7–64.9)
JC virus, %	9	22.5	19	12.5
Log (JC titers)^a^	2.78	4.4 (3.7–5.5)	4.3 (3.2–5.5)	2.9 (2.3–4.9)
BK virus, %	27.2	32.2	30.9	17.5
Log (BK titers)^a^	1.27 (1–1.9)	3.8 (2.4–4.6)	3.3 (1.2–4.6)	2.3 (1.8–3)
HIV virus, %	54.5	19.3	28.5	37.5
Log (HIV titers)^a^	1.6 (1.5–3.6)	3 (1.9–4.2)	2.2 (1.5–4.3)	3.2 (2–4.3)
	**HIV+** **controls without CKD**			
	**APOL1 -**	**APOL1 -**	**APOL1 -**	**APOL1 -**
	**0 risk alleles (39)**	**1 risk alleles (51)**	**0** **+** **1 risk alleles (90)**	**2 risk alleles (27)**
Age at enrollment, y	39.2 (33.9–47)	38.9 (34.6–49.9)	39.1 (34.2–49.8)	43.9 (38.2–48.8)
Female sex, *n* (%)	76.9	72.5	74.4	62.9
CKD-EPI eGFR, mL/min/1.73 m^2^	117.8 (104.4–131.8)	124.6 (109–130.86)	121.6 (106.6–131.6)	120 (109.5–130.3)
UACR, mg/mmol	0.6 (0.5–1.2)	0.6 (0.4–1)	0.6 (0.4–1)	0.7 (0.5–1)
JC virus, %	17.9	25.5	22.2	7.4
Log (JC titers)^a^	4.6 (2.6–5.3)	5.1 (3.3–5.8)	4.7 (2.7–5.8)	4.7 (4.2–5.2)
BK virus, %	10.2	31.3	22.2	18.5
Log (BK titers)^a^	1.5 (1.1–1.6)	2.6 (1.2–3.7)	2 (1.1–3.5)	1 (1–3.1)
HIV virus, %^b^	15.3	27.4	22.2	51.84
Log (HIV titers)^a^	2.5 (2.3–2.8)	2.2 (1.5–2.8)	2.2 (1.6–2.9)	3.2 (1.7–4.8)

*Data presented as median (25th percentile to 75th percentile) for continuous variables or % for categorical variables*.

*CKD-EPI, Chronic Kidney Disease Epidemiology Collaboration*.

a *Data from subjects that have viremia or viruria measured*.

b *HIV viremia: more than 20 copies/ml*.

**Table 3:** Odds ratio (OR) and 95% confidence intervals (CIs) for the association with CKD.

	**CKD OR (95% CI)**	***P*-value**
Age	1.02 (0.99–1.06)	0.09
Gender	0.77 (0.39–1.5)	0.45
APOL1 1 RRA	4.42 (1.491–13.158)	0.007
APOL1 2 RRA	12.6 (3.89–40.80)	<0.0001
HIV viremia	7.45 (1.66–33.35)	0.009
BK viruria	1.17 (0.57–2.42)	0.65
JC viruria	0.84 (0.36–1.94)	0.68
APOL1 1 RRA* HIV viremia^a^	0.081 (0.01–0.52)	0.008
APOL1 2 RRA* HIV viremia^a^	0.073 (0.01–0.45)	0.005

a *APOL1 RRA interaction with HIV viremia*.

**Table 4:** Odds ratio (OR) and 95% confidence intervals (CIs) for the association between *APOL1* risk genotype and CKD.

**APOL RRA**	**Model 1, OR**	**Model 2, OR**	**Model 3, OR**	**Model 4, OR**
	**(95% CI)**	**(95% CI)**	**(95% CI)**	**(95% CI)**
1	2.07 (0.92–4.66)	2.03 (0.89–4.63)	2.03 (0.89–4.63)	4.42 (1.49–13.15)
2	5 (2.17–11.547)	4.96 (2.15–11.46)	4.96 (2.15–11.46)	12.6 (3.89–40.8)

Multivariate logistic regression with stepwise and forward model selection was used to predict the risk for CKD. The explanatory variable is number of APOL1 risk alleles: model 1, adjusted for age and sex; model 2, further adjusted for JC and BK viruria; model 3 further adjusted for HIV viremia, model 4 further adjusted for APOL1 RRA^*^ HIV interaction.

### Prevalence of HIV Viremia

Participants with CKD (cases) and those without CKD (controls) were evaluated for HIV viral load, JCV and BKV viruria. All HIV-positive patients recruited from HIV clinics were likely to have been initiated on antiretroviral therapy (ART) before recruitment into the study. To determine whether *APOL1* G1 or G2 RRAs affect host susceptibility to HIV viremia, multivariate logistic regression with a stepwise, forward and backward model selection was used to predict HIV viral load. Variables included in the model were age, gender, JC viruria, BK viruria, *APOL1* RRA (2 vs. 1 vs. 0), CKD, e GFR and urine albumin to creatinine ratio. The two *APOL1* RRA genotypes were associated with an increased probability of having HIV viremia OR 2.37 (95% CI 1.0–5.63, *p* = 0.05) (Table 5). Moreover, a higher viral load (more than 40,000 copies/ml) was observed in patients with 2 RRA compared with 0, 1 RRA OR 7.583 (95% CI 1.530–37.597, *p* = 0.013). Interestingly, males were found to have a higher risk of HIV viral load compared to females (>40,000/ml; OR of 4.552, 95% CI 1.1–18.829, *p* = 0.036). While previous studies have pointed to reduced ART success in males, that only partially is attributable to reduced ART adherence in males compared to females (24), the current study does not include information about ART adherence that could potentially explain this difference.

**Table 5:** Odds ratio (OR) and 95% confidence intervals (CIs) for the association with JC and BK viruria and HIV viremia.

	**JC viruria OR**	***P*-value**	**BK viruria OR**	***P*-value**	**HIV viremia OR**	***P*-value**
	**(95% CI)**		**(95% CI)**		**(95% CI)**	
Age	1.035 (0.992–1.080)	0.111	0.993 (0.955–1.032)	0.720	0.994 (0.96–1.03)	0.749
Gender	1.469 (0.606–3.56)	0.395	0.962 (0.437–2.117)	0.923	0.809 (0.401–1.63)	0.553
CKD	1.162 (0.144–9.356)	0.888	6.958 (1.003–48.258)	0.050	0.519 (0.09–2.977)	0.462
eGFR	1.005 (0.982–1.029)	0.655	1.017 (0.996–1.038)	0.108	0.992 (0.975–1.01)	0.408
Urine Albumin/creatinine	1.002 (0.997–1.006)	0.426	0.988 (0.967–1.009)	0.265	1.001 (0.998–1.003)	0.535
APOL		0.333		0.068		0.067
APOL1 1 risk variant	1.645 (0.63–4.297)	0.309	2.33 (0.934–5.81)	0.070	1.098 (0.466–2.582)	0.831
APOL1 2 risk variants	0.841 (0.26–2.716)	0.772	1.004 (0.341–2.957)	0.994	2.375 (1.001–5.635)	0.050
BK viruria	1.156 (0.47–2.841)	0.752			1.303 (0.6–2.83)	0.503
HIV viremia	0.351 (0.125–0.982)	0.046	1.308 (0.602–2.839)	0.498		
JC viruria			1.154 (0.47–2.836)	0.754	0.345 (0.122, 0.98)	0.046

### Association of BK and JC Viruria

HIV viremia was associated with a significant effect on the probability of JC virus urine shedding, reducing the probability of JC viruria compared to individuals without HIV viremia OR 0.35 (95% CI 0.12–0.98, *P* = 0.046; Table 5). JC viruria did not differ between patients with CKD and controls (*p*-value = 0.67). In addition, subjects with *APOL*1 RRA who had JC viruria did not display a lower prevalence of CKD compared to individuals who did not have JC viruria (*p*-value = 0.692). BK viruria was not associated with CKD and HIV viremia (*p*-value 0.65 and 0.503, respectively). In light of previous publications about the inhibitory interaction between BK and JC viruria (25), we assessed this possibility. As we previously reported (14), there was no correlation between BK and JC viruria (Pearson correlation coefficient: 0.43, p=0.23).

## Discussion

In this genetic epidemiological study of HIV infected individuals living in Ghana and Nigeria (26), we observed an association between 2 *APOL1* RRA and HIV viremia and CKD. Notably, there was a trend for increased CKD risk with one *APOL1* RRA that was significantly increased after adjustment for APOL1 RRA^*^HIV viremia interaction. This increased risk is in keeping with the reported marginal association of HIVAN with 1 risk allele, in individuals of black African ancestry from South Africa (7). Similarly, previous studies have also reported a marginal effect of one *APOL1* RRA (1, 2, 27, 28), suggesting that the possibility that an environment (HIV)-gene interaction may enhance the risk of CKD, in carriers of one *APOL1* RRA. Therefore, HIV is a robust risk factor for kidney disease, that may potentiate the genetic risk, even in individuals with only one risk allele, into manifest kidney disease. Innate immune responses to viruses can drive APOL1 kidney disease in patients with *APOL1* high-risk genotypes as was demonstrated in case series of collapsing glomerulopathy caused by therapeutic administration of interferon products and in patients infected with COVID-19 (29, 30). Interestingly, one *APOL1* RRA in kidney allograft was associated with collapsing glomerulopathy in a patient infected with COVID-19 (30), suggesting that one *APOL1* risk allele may confer a pathogenic role in diseases that share enhanced activation of the innate immune system. For completeness, we point out that in a combined interaction analysis, the interaction of HIV viremia and 2 APOL1 RRA was associated with decreased CKD risk. We consider this not to reflect a biological effect but rather a statistical effect that can occur in interaction analyses of separately contributory associations, termed Simpson's paradox, but still make note in case future biological or clinical research is warranted to explain this result (21–23).

In addition, we demonstrate a complex interaction of HIV viremia with polyoma virus urine shedding. The increased HIV viral load was associated with reduced JC viruria, while BK viruria was not associated with CKD or HIV viremia. We did not demonstrate the protective association of JC viruria with preserved kidney function, as previously reported in patients who did not have HIV infection and had non-diabetic as well as diabetic kidney disease (12–14). We reasoned that in the presence of HIV viremia, the secretion of inflammatory cytokines, such as Interferon itself, might lead to restriction of other viruses, including suppression of JC viruria and thereby mask any such association.

The role of APOL1 as a member of the innate immune system is only partially understood. APOL1 has a well-characterized protective role that restores trypanolysis activity against *Trypanosoma brucei rhodesiense* (G2) and confers protection from active illness caused by *Trypanosoma brucei gambiense* (G1) (1, 4–6). It is not clear if APOL1 has broader protective roles as an innate immunity protein. Most interestingly, a recent analysis of the African-American participants of the Reasons for Geographic and Racial Differences in Stroke (REGARDS) study, demonstrated that *APOL1* RRA were associated with sepsis risk under dominant [Hazard Ratio (HR) 1.55, 95% confidence interval (CI), 1.13–2.11] and additive (HR per variant allele copy 1.25, 95% CI, 1.02–1.53) genetic models adjusted for covariates and ancestry, suggesting a complex interaction between *APOL1* and general risk for severe infection (31). In contrast to the increased sepsis risk, some *in vitro* studies have demonstrated that APOL1 restricts HIV infectivity and protects human keratinocytes from the lethal effect of the Staphylococcus aureus α toxin (8, 9, 32). Specifically, impaired HIV restriction in podocytes by the APOL1 encoded by RRA, has been suggested (10). However, an epidemiological study did not detect an association between HIV viral load and *APOL1* variants. The ALIVE cohort revealed no evidence of association of *APOL1* RRA with HIV infection acquisition, viral load or disease progression, in ART naïve patients (11). In contrast, the current study of ART treated patients points to increased risk for HIV viremia in association with certain *APOL1* genotypes. In accordance to the World Health Organization and International Antiviral Society-USA Panel recommendations from 2015 for early ART initiation regardless of CD4 cell-count (33), all participants were likely to have received ART, but we cannot confirm adherence. To the best of our knowledge, this is the first observed significant association between *APOL1* risk variants and HIV viremia in patients receiving ART. The complex interaction between virus, host, treatment status and environmental factors may explain this association in contrast to previous publications. The dominant HIV subtype is different in West Africa vs. South Africa and the Americas (CRF02_AG, subtype G vs. C and B, respectively) (34, 35). Different subtypes may have different biological properties resulting in differences in transmissibility and pathogenicity. In addition, the participants of the current cohort were treated with ART although adherence was not established. This is in contrast to the ALIVE cohort that was a prospective longitudinal natural cohort originally designed to characterize the incidence and natural history of HIV infection. Moreover, in the ALIVE study, viral loads not reflecting the steady-state (viral load measurements exceeding 3-fold from the average of all remaining points) were excluded (11), thereby, reducing the probability of detecting transient viremia. Given the absence of longitudinal follow up in our cohort, we cannot determine whether the viremia detected was transient. In addition, we do not have data about CD4 cell count in parallel with HIV viral load. Similarly, we cannot determine whether these findings impact clinical outcomes, such as incidence of CKD as well as other HIV outcomes.

We demonstrate complex interactions between HIV viremia, CKD and polyoma viruria. Previous studies reported reduced JC viruria in CKD patients (12–14). Herein, reduced JC viruria was driven by HIV viremia and not CKD. We postulated that the reduced JC viruria in kidney disease and the presence of HIV viremia share similar signaling pathways that enhance the activation of the innate immune system. Enhanced inflammatory signaling (e.g., via the type I interferon signaling cascade that abrogates JC replication) restricts JCV growth and leads to decreased shedding of JCV (14, 36). Indeed, *in vitro* studies have demonstrated divergent interaction between IFN-β and the two polyoma viruses. IFN-β controls JCV replication but fails to restrict BKV (36), thereby, explaining the specific restriction of JC virus shedding in urine and not BK in patients with HIV viremia. We hypothesize that absence of JC viruria is an epiphenomenon, serving as a biomarker that reflects innate immune system activation. In contrast to JC viruria, only BK viruria is enhanced in immunocompromised patients with HIV infection and correlates inversely with the CD4+ cell count (15–17). In the current cohort, BK viruria was not associated with CKD and HIV viremia. Most BK virus associated nephropathy occurs in renal allograft patients after kidney transplantation. However, some case reports have described BK virus-associated nephropathy in the native kidney, particularly in patients with human immunodeficiency virus infection (37, 38). Further studies are needed to explore the role of BK viruria in CKD patients infected with HIV.

The present study has limitations: This cohort has a relatively small sample size. HIV viral load was measured only at enrollment, so we could not determine if the enhanced viral load was transient or persistent. Similarly, we do not have information about CD4 cells count, ART compliance and longitudinal clinical outcomes of kidney function were missing. Therefore, we could not ascertain if the increased HIV viremia will translate to faster CKD progression, as was previously demonstrated (39). In addition, JC and BK viruria may fluctuate and was measured at a single time point over time in the current study.

In conclusion, this population genetic study demonstrates an increased risk for HIV viremia and CKD in patients with one and two *APOL1* renal risk alleles. The association of one *APOL1* risk allele with CKD suggests that if G0 has a protective role, it does not have complete suppressive penetrance; alternatively, this finding is also consistent with a gain of injury mechanism mediating kidney injury in individuals with a robust environmental insult, such as HIV infection. Future studies are warranted to explore the complex interaction of *APOL1* RRA, CKD risk, HIV viremia and urine shedding of polyoma viruses. Confirmation in future studies of an association between two *APOL1* risk alleles with HIV viremia and CKD would have significant therapeutic implications regarding APOL1 inhibition in patients with two risk alleles, who do not reside in Trypanosoma endemic areas.

## Data Availability Statement

The original contributions presented in the study are included in the article/supplementary material, further inquiries can be directed to the corresponding author/s.

## Ethics Statement

The studies involving human participants were reviewed and approved by University of Ghana (Ghana Health Service Ethics Review Committee). The patients/participants provided their written informed consent to participate in this study.

## Author Contributions

EK-D, BMS, AG, AN, GA, BLS, AO, DA, and KS contributed to conception and design of the study. AG, AN, and GA supervised the study. IU, YR, VB, CO, MMat, TO, SAj, MMam, VB, SAn, AA, JP-R, VMA, and FA enrolled patients. EK-D, BMS, and DA organized the database. EK-D, KS, BMS, CW, AO, and DA wrote the draft of the manuscript. MC performed the polyoma viruses analysis. SAg performed the HIV viral load analysis. AR and BH performed the statistical analysis. BMS, EA-A, PA, and CW performed the genotype analysis. All authors contributed to manuscript revision, read, and approved the submitted version.

## Conflict of Interest

KS – Inventor on patent USTPO 77864 entitled: Methods and Kits for Determining Predisposition to Kidney Disease. The remaining authors declare that the research was conducted in the absence of any commercial or financial relationships that could be construed as a potential conflict of interest.

## Publisher's Note

All claims expressed in this article are solely those of the authors and do not necessarily represent those of their affiliated organizations, or those of the publisher, the editors and the reviewers. Any product that may be evaluated in this article, or claim that may be made by its manufacturer, is not guaranteed or endorsed by the publisher.
